# Rhizobacteria and Arbuscular Mycorrhizal Fungi of Oil Crops (Physic Nut and Sacha Inchi): A Cultivable-Based Assessment for Abundance, Diversity, and Plant Growth-Promoting Potentials

**DOI:** 10.3390/plants9121773

**Published:** 2020-12-14

**Authors:** Janjira Wiriya, Chakrapong Rangjaroen, Neung Teaumroong, Rungroch Sungthong, Saisamorn Lumyong

**Affiliations:** 1 Department of Biology, Faculty of Science, Chiang Mai University, Chiang Mai 50200, Thailand; janjira.wiriya209@gmail.com; 2 Graduate School, Chiang Mai University, Chiang Mai 50200, Thailand; 3 Department of Agricultural Management Technology, Faculty of Science and Technology, Phranakhon Rajabhat University, Bangkok 10220, Thailand; rangjaroen@gmail.com; 4 School of Biotechnology, Institute of Agricultural Technology, Suranaree University of Technology, Nakhon Ratchasima 30000, Thailand; neung@sut.ac.th; 5 Laboratory of Hydrology and Geochemistry of Strasbourg, University of Strasbourg, UMR 7517 CNRS/EOST, Strasbourg CEDEX 67084, France; 6 Center of Excellence in Microbial Diversity and Sustainable Utilization, Faculty of Science, Chiang Mai University, Chiang Mai 50200, Thailand; 7 Academy of Science, The Royal Society of Thailand, Bangkok 10300, Thailand

**Keywords:** oil crop, physic nut, sacha inchi, rhizobacteria, arbuscular mycorrhizal fungi, plant growth-promoting activity

## Abstract

Nowadays, oil crops are very attractive both for human consumption and biodiesel production; however, little is known about their commensal rhizosphere microbes. In this study, rhizosphere samples were collected from physic nut and sacha inchi plants grown in several areas of Thailand. Rhizobacteria, cultivable in nitrogen-free media, and arbuscular mycorrhizal (AM) fungi were isolated and examined for abundance, diversity, and plant growth-promoting activities (indole-3-acetic acid (IAA) and siderophore production, nitrogen fixation, and phosphate solubilization). Results showed that only the AM spore amount was affected by plant species and soil features. Considering rhizobacterial diversity, two classes—*Alphaproteobacteria* (*Ensifer* sp. and *Agrobacterium* sp.) and *Gammaproteobacteria* (*Raoultella* sp. and *Pseudomonas* spp.)—were identified in physic nut rhizosphere, and three classes; *Actinobacteria* (*Microbacterium* sp.), *Betaproteobacteria* (*Burkholderia* sp.) and *Gammaproteobacteria* (*Pantoea* sp.) were identified in the sacha inchi rhizosphere. Considering AM fungal diversity, four genera were identified (*Acaulospora*, *Claroideoglomus*, *Glomus*, and *Funneliformis*) in sacha inchi rhizospheres and two genera (*Acaulospora* and *Glomus*) in physic nut rhizospheres. The rhizobacteria with the highest IAA production and AM spores with the highest root-colonizing ability were identified, and the best ones (*Ensifer* sp. CM1-RB003 and *Acaulospora* sp. CM2-AMA3 for physic nut, and *Pantoea* sp. CR1-RB056 and *Funneliformis* sp. CR2-AMF1 for sacha inchi) were evaluated in pot experiments alone and in a consortium in comparison with a non-inoculated control. The microbial treatments increased the length and the diameter of stems and the chlorophyll content in both the crops. CM1-RB003 and CR1-RB056 also increased the number of leaves in sacha inchi. Interestingly, in physic nut, the consortium increased AM fungal root colonization and the numbers of offspring AM spores in comparison with those observed in sacha inchi. Our findings proved that AM fungal abundance and diversity likely rely on plant species and soil features. In addition, pot experiments showed that rhizosphere microorganisms were the key players in the development and growth of physic nut and sacha inchi.

## 1. Introduction

**Figure 1:**
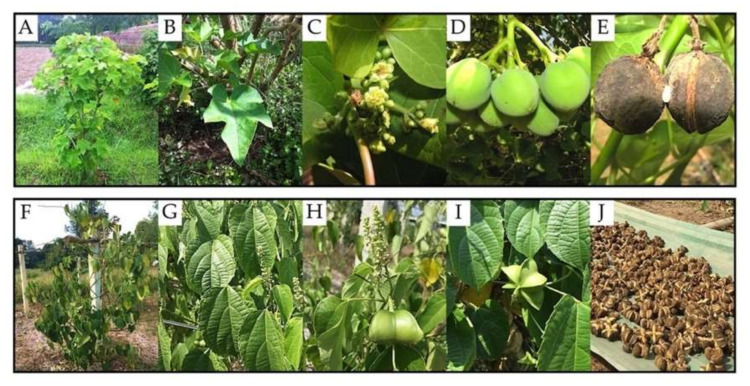
Morphological characteristics of physic nut (**A**–**E**) and sacha inchi (**F**–**J**). The images show features of stems and branches (**A**,**F**), leaves (**B**,**G**), flowers (**C**,**H**), young fruits (**D**,**I**), and mature fruits (**E**,**J**).

Physic nut (*Jatropha curcas* L.) and sacha inchi (*Plukenetia volubilis* L.) (Figure 1) belong to the same family, *Euphorbiaceae*, but different subfamilies—*Crotonoideae* for physic nut and *Acalyphoideae* for sacha inchi. Physic nut is a medium-sized shrub with a height of ~6 m and a reproductive life of up to 50 years [1], and it is well considered an economic plant as a source of biofuel [2,3,4,5,6]. This plant is also capable of growing in variable climatic conditions, areas with nutrient deficiency, and arid soil with <40% plant-available water [7,8,9,10,11]. The commercial interest in the plant and its ability to grow in hard areas make physic nut a good crop for enhancing the economy in a sustainable way. Sacha inchi, known as “sacha peanut, mountain peanut, or Inca peanut,” is a native species of South America and is also being increasingly cultivated in northern Thailand and the Greater Mekong Sub-region [12]. This crop is produced mainly for human consumption rather than bioenergy purposes as its seeds are rich in protein, fatty acids (e.g., omega-3 and omega-6), and oil (30–60%) [12,13,14]. Sacha inchi oil has been renowned mostly for its nutritive and pharmaceutical values [12,13,14], and only a few studies reveal its potential for biofuel production [15,16].

A plant’s rhizosphere is well recognized as an energetic zone where the plant communicates and interacts with abundant microbes using phytochemicals in the form of root exudates [17,18,19,20,21,22,23,24]. Among soil microbes, commensal rhizosphere microbes, such as plant growth-promoting rhizobacteria (PGPR) and arbuscular mycorrhizal (AM) fungi, play a key role in the development and growth of crops [17,18,19,20,21,22,23,24]. The keystone functions of PGPR include, but are not limited to, the synthesis of phytohormones (e.g., indole-3-acetic acid (IAA), gibberellic acid, and cytokinin) [25,26,27,28], the supply of plant nutrients (e.g., nitrogen via fixation, iron via siderophore production, and phosphorus via solubilization) [29,30,31,32,33], etc. AM fungi can enhance nutrient uptake of several host plants [34,35,36,37,38,39]. Some AM fungi are able to increase plant tolerance to abiotic and biotic stresses [40,41,42,43,44]. Many research studies investigating the combined use of AM fungi with other rhizosphere organisms (e.g., PGPR, earthworm) showed synergic effects of these consortia on plant growth and development [45,46,47]. However, some unfavorable combinations were also found. For example, Svenningsen et al. [48] reported that *Acidobacteria* and other fungal antagonists could suppress the ability of AM fungi to improve plant uptake of phosphorus.

Relatively few studies have elucidated the community structures and/or plant growth-promoting traits of bacteria [49,50,51,52,53,54], AM fungi [10,55,56,57], or both [58,59] in physic nut rhizospheres, and many of these studies have not reported the origin of microorganisms (such as rhizosphere, soil, root, etc.). For sacha inchi, to our knowledge, little is known about the commensal rhizosphere microbes of this plant species. Some studies recently discovered novel AM species that live associated with sacha inchi rhizospheres [60,61,62]. Only one report demonstrates the genetic diversity of rhizosphere microbes (i.e., bacteria and fungi) of sacha inchi cultivated in different land-use conversion soils [63], and another one assesses the interactions between AM fungi and sacha inchi roots in promoting the growth and development of this plant under drought conditions [64]. With the limited plant-microbe interaction data on these oil crops, further investigations are yet required to fill any remaining knowledge gaps. For instance, can the geographical locations and soil physicochemical properties affect the microbial structures of oil crop rhizospheres? How do rhizosphere microbial structures differ from oil crop to oil crop? What are the principal microbial taxa dwelling in oil crop rhizospheres, and how do they contribute to plant growth and development? Can beneficial rhizosphere microbes of oil crops be applied as alternative plant enhancers in the sustainable agriculture for oil crop production? 

In this study, we aim to investigate the abundance and diversity of viable bacteria and AM fungi dwelling in physic nut and sacha inchi rhizospheres. The impacts of plant species, geographical locations, and soil physicochemical properties on the abundance and diversity of rhizosphere microbes derived from both plants are addressed and discussed. All isolated rhizosphere microbes were evaluated for their plant growth-promoting activities or ability to colonize plant roots. Some rhizobacteria and AM fungi were selected to verify their individual or mutual capacity to be applied further as effective plant biostimulants in pot experiments. 

## 2. Results

### 2.1. The Abundance of Physic Nut and Sacha Inchi Rhizosphere Microbes

**Figure 2:**
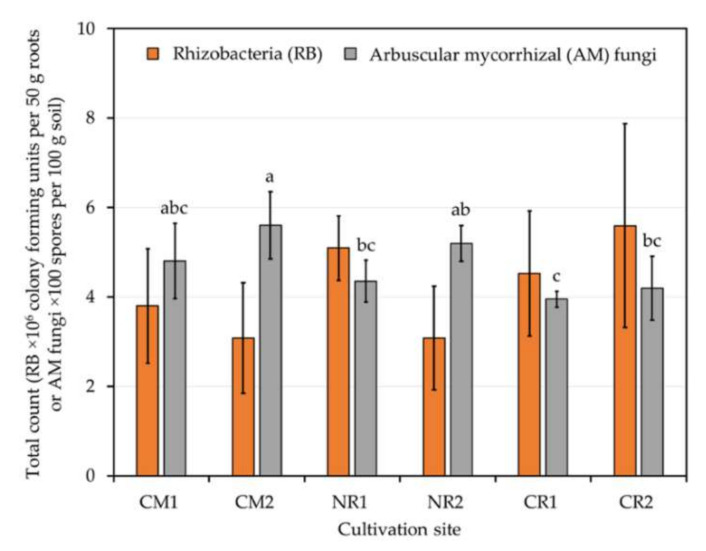
Total counts of rhizobacteria and arbuscular mycorrhizal (AM) spores residing in the rhizospheres of physic nut and sacha inchi grown in different cultivation sites. Every count was done at least in duplicate. Information about cultivation sites and host plants is available in Table 1, and the different lower-case letters refer to the statistical difference analyzed by one-way ANOVA (*F*_(5, 24)_ = 5.44, *p* = 0.002). No significant difference was observed for rhizobacterial counts across cultivation sites (*F*_(5, 7)_ = 1.10, *p* = 0.437).

The total counts of rhizobacteria dwelling on the surfaces of 50 g roots of physic nut or sacha inchi grown at different geographical locations (Table 1) were in a range of 10^6^ colony-forming units (CFUs) (Figure 2). These bacterial counts did not differ significantly, regardless of distinct plant species and cultivation sites. Significant differences were observed for the number of AM spores (Figure 2). The highest numbers were found in the rhizospheres of physic nut plants grown at CM2. On the other hand, the lowest values were found in the rhizospheres of sacha inchi plants grown at CR1.

### 2.2. Plant Growth-Promoting Activities of Rhizobacteria and Their Identification

**Figure 3:**
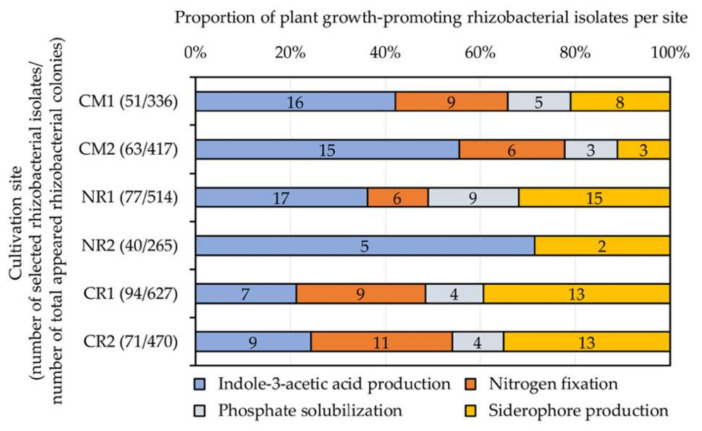
Proportions of plant growth-promoting rhizobacteria isolated from different cultivation sites of physic nut (CM1, CM2, NR1, and NR2) and sacha inchi (CR1 and CR2). The rhizobacteria included in these tests were randomly selected based on their colony features, accounting for 15% of all appeared bacterial colonies per site. Numerical data indicated in the bar graphs refer to the numbers of rhizobacterial isolates giving positive tests. Information about cultivation sites and host plants is available in Table 1.

A total of 396 selected rhizobacterial isolates (Table 2) were assessed for their plant growth-promoting activities (Figure 3). These tested rhizobacteria were randomly selected based on their colony features, accounting for 15% of all appeared bacterial colonies per site. IAA-like molecule- and siderophore-forming, nitrogen-fixing, and phosphate-solubilizing rhizobacteria were found at every site, except for NR2, where only nitrogen-fixing and phosphate-solubilizing rhizobacteria were found (Figure 3). The proportions of rhizobacteria possessing different plant growth-promoting activities were similar between sites in the same geographical location (i.e., CM1 and CM2 in Chiang Mai or CR1 and CR2 in Chiang Rai), except for those observed in Nakhon Ratchasima (NR1 and NR2) (Figure 3). Physic nut rhizospheres at NR1 housed the highest numbers of IAA-forming (17 isolates), siderophore-forming (15 isolates), and phosphate-solubilizing (9 isolates) rhizobacteria. The highest number of nitrogen-fixing rhizobacteria (11 isolates) was found in the rhizospheres of sacha inchi grown at CR2.

**Figure 4:**
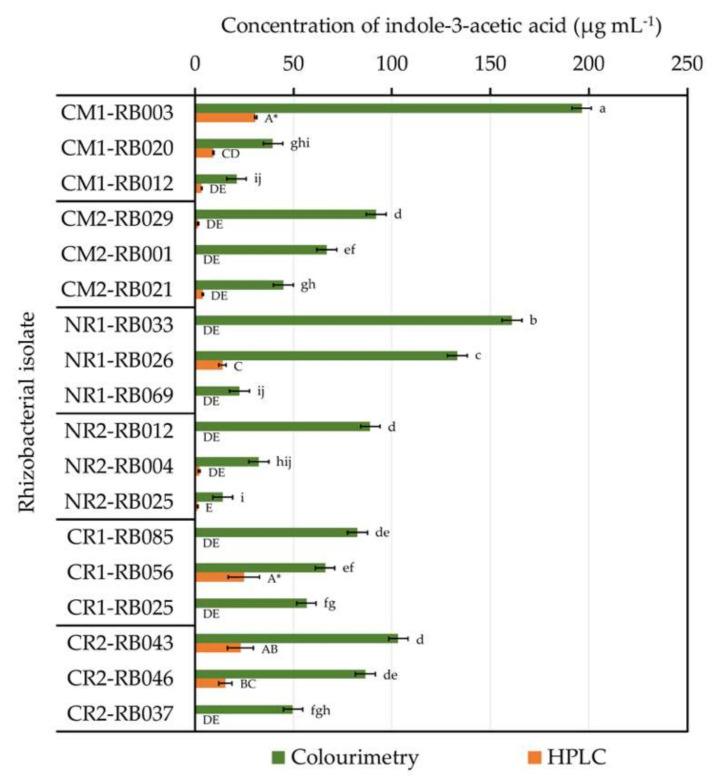
The capability of physic nut and sacha inchi rhizobacteria to produce indole-3-acetic acid (IAA). Only the top three excellent IAA-forming isolates per cultivation site are presented. The IAA-produced concentrations were primarily estimated with colorimetry and were consequently quantified by high-performance liquid chromatography (HPLC). Every measurement was done in triplicate. The origins of these bacteria are available in Table 2. The difference of lower-case letters or capital letters refers to the statistical difference by one-way ANOVA across results derived from colorimetry (*F*_(17, 36)_ = 156.06, *p* ≤ 0.0005) or HPLC (*F*_(17, 36)_ = 44.57, *p* ≤ 0.0005), respectively. Asterisks refer to the rhizobacterial isolates that produced the highest level of IAA assessed by HPLC.

Among all tested rhizobacteria, isolates CM1-RB002, CM1-RB013, NR1-RB010, and NR1-RB028 exhibited every plant growth-promoting activity assayed in vitro, but the values observed were relatively low in comparison with other isolates (data not shown). The best three rhizobacteria (per cultivation site) that produced the highest content of IAA-like molecules by the colorimetric test were also assessed by high-performance liquid chromatography (HPLC) analysis to precisely elucidate their ability to form IAA (Figure 4). The best three rhizobacteria for other plant growth-promoting activities are listed in the Supplementary Materials, Figure S1.

**Figure 5:**
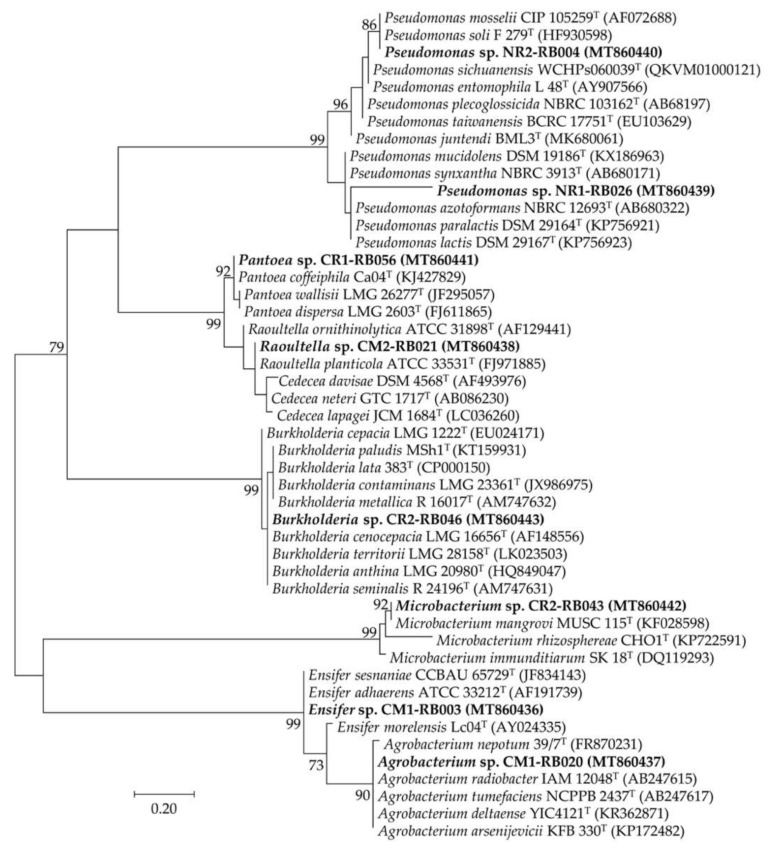
Unrooted maximum likelihood phylogenetic tree of promising plant growth-promoting rhizobacteria isolated from physic nut and sacha inchi rhizospheres. The origins of these bacteria are available in Table 1 and Table 2. The tree was constructed using 16S rRNA gene sequence data derived from our selected isolates (in bold) and their closely related phylogenetic species. The GenBank accession number of the gene sequence is presented in the parenthesis. Bootstrap values (based on 1000 replications) of > 70% are at the tree’s nodes, and the scale bar represents 20% dissimilarity.

Using HPLC-based IAA-producing activity to determine the best rhizobacterial performance, isolate CM1-RB003 was the best IAA-forming rhizobacterium, followed by isolate CR1-RB056 (Figure 4 and Table 3). The excellent IAA-forming rhizobacteria (Table 3) were chosen for genotypic identification using their 16S rRNA gene sequence data (Table 4 and Figure 5). The selected rhizobacteria of physic nut belonged to two classes, *Alphaproteobacteria* (*Ensifer* sp. CM1-RB003 and *Agrobacterium* sp. CM1-RB020) and *Gammaproteobacteria* (*Raoultella* sp. CM2-RB021 and *Pseudomonas* spp. NR1-RB026 and NR2-RB004). Conversely, the best IAA-forming rhizobacteria isolated from sacha inchi belonged to three classes, *Actinobacteria* (*Microbacterium* sp. CR2-RB043), *Betaproteobacteria* (*Burkholderia* sp. CR2-RB046), and *Gammaproteobacteria* (*Pantoea* sp. CR1-RB056).

### 2.3. The Diversity and Proliferation of AM Fungi and Their Identification

The data on AM spore abundance are shown in Figure 1 and Table 5. The spores were classified in different groups based on morphological features. A total of nine groups of the genus *Acaulospora* (A1–A9), a group of the genus *Claroideoglomus* (C1), two groups of the genus *Glomus* (G1 and G2), and a group of the genus *Funneliformis* (F1) were found (Table 5 and Figure S2).

**Figure 6:**
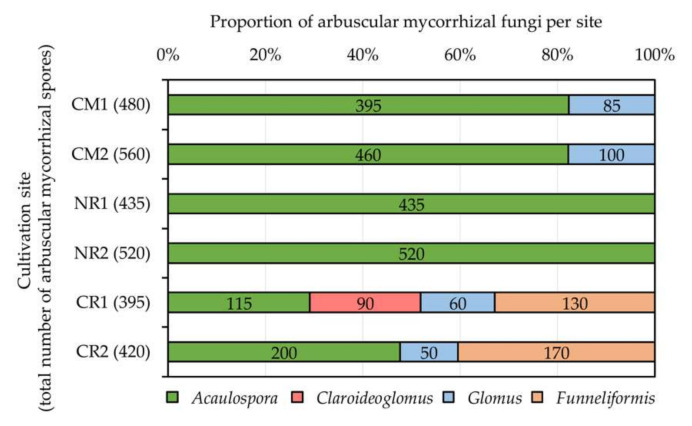
Proportions of arbuscular mycorrhizal (AM) fungi assessed at different cultivation sites of physic nut (CM1, CM2, NR1, and NR2) and sacha inchi (CR1 and CR2). Numerical data indicated in the bar graphs refer to the numbers of AM spores belonging to each AM genus. Information about the cultivation sites and host plants is available in Table 1.

The proportion of AM genera per cultivation site is presented in Figure 6. Sacha inchi rhizospheres at CR1 housed the most diverse AM fungi, including *Acaulospora*, *Claroideoglomus*, *Glomus*, and *Funneliformis*. Only one AM genus, *Acaulospora*, was detected in physic nut rhizospheres grown at NR1 and NR2. The genus *Acaulospora* was the predominant AM fungus found in most cultivation sites explored, except for CR1, where *Funneliformis* was the dominant genus. Moreover, the genus *Claroideoglomus* was found only in sacha inchi rhizospheres grown at CR1.

**Figure 7:**
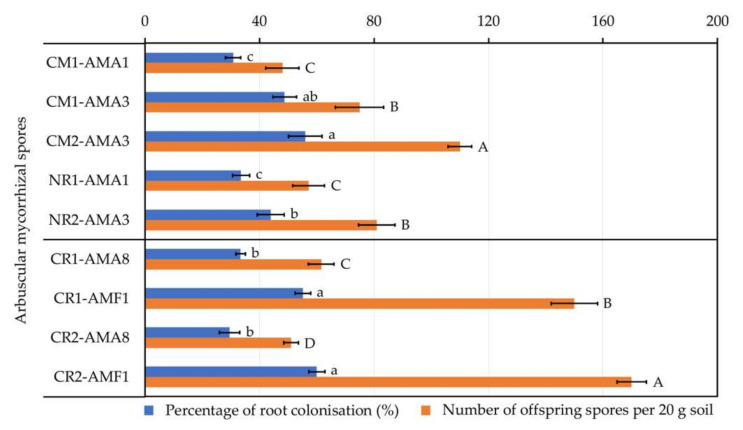
The proliferation of arbuscular mycorrhizal (AM) fungi derived from rhizospheres of physic nut (coding with CM1, CM2, NR1, and NR2) and sacha inchi (coding with CR1 and CR2). Only AM spores that could colonize plant roots and produce offspring spores are presented. Every assessment was done in five replicates. The origins of these AM spores are available in Table 5. The difference in lower-case letters or capital letters refers to the statistical difference of means compared by one-way ANOVA across the percentages of root colonization (*F*_(4, 20)_ = 30.84, *p* ≤ 0.0005 for physic nut and *F*_(3, 16)_ = 151.50, *p* ≤ 0.0005 for sacha inchi) or numbers of offspring spores per 20 g soil (*F*_(4, 20)_ = 74.34, *p* ≤ 0.0005 for physic nut and *F*_(3, 16)_ = 623.08, *p* ≤ 0.0005 for sacha inchi), respectively.

AM spores belonging to each morphological group per each cultivation site (Table 5) were tested for their proliferation in association with their corresponding host plants, and only AM spores that could colonize plant roots and produce offspring spores were compared statistically (Figure 7). For physic nut, AM spore CM2-AMA3 exhibited both the highest percentage of plant root colonization (56.00 ± 5.23%) and the highest number of offspring spores produced (110 ± 4.18 spores per 20 g soil). On the other hand, for sacha inchi, AM spores CR1-AMF1 and CR2-AMF1 showed the highest percentages of root colonization (55.20 ± 2.68% and 60.00 ± 2.83%, respectively) and AM spore CR2-AMF1 produced the highest number of offspring spores (170 ± 5.10 spores per 20 g soil). 

**Figure 8:**
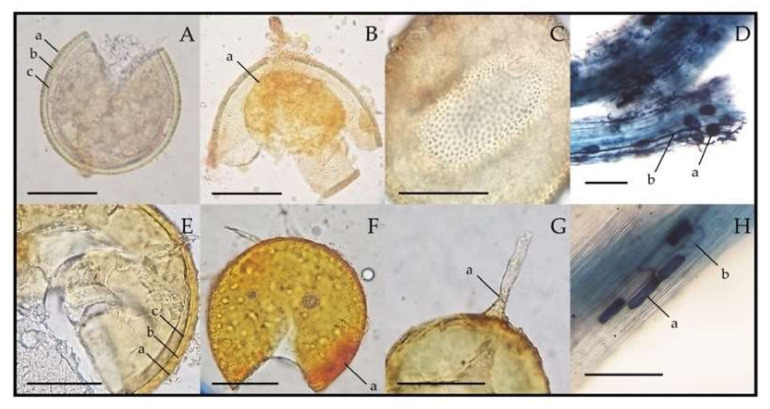
Spore features and root colonizations of the most prolific arbuscular mycorrhizal (AM) fungi derived from the rhizosphere soils of physic nut (*Acaulospora* sp. CM2-AMA3, (**A**–**D**)) and sacha inchi (*Funneliformis* sp. CR2-AMF1, (**E**–**H**)). In (**A**) and (**E**), a = layer 1, b = layer 2, and c = layer 3 of spore wall. (**B**) and (**F**) show crushed spore stained with Melzer’s reagent, where a = germination wall (**B**) and a = spore wall (**F**). (**C**) shows the surface of the spore wall covered with excrescences or beads. In (**G**), a = funnel-shaped base of subtending hypha. In (**D**) and (**H**), a = vesicles and b = hyphae inside physic nut (**D**) and sacha inchi (**H**) roots. Scale bars = 100 µm.

**Figure 9:**
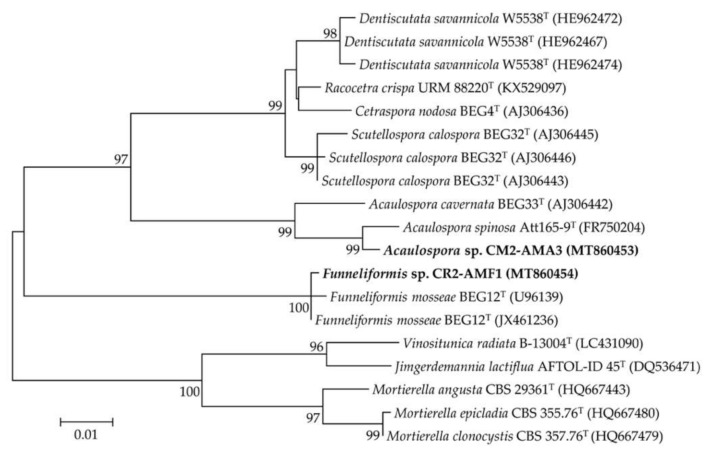
Unrooted maximum likelihood phylogenetic tree of the most prolific arbuscular mycorrhizal (AM) fungi derived from physic nut and sacha inchi rhizospheres. The origins of these AM fungi are available in Table 1 and Table 5. The tree was constructed using 18S rRNA gene sequence data derived from our selected AM spores (in bold) and their closely related phylogenetic species. The GenBank accession number of the gene sequence is presented in the parenthesis. Bootstrap values (based on 1000 replications) of > 70% are at the tree’s nodes, and the scale bar represents 1% dissimilarity.

The most prolific AM spores CM2-AMA3 and CR2-AMF1 belonged to the morphological groups of the genera *Acaulospora* and *Funneliformis*, respectively (Table 5). The morphological characteristics of their spores and root colonization are shown in Figure 8. Both CM2-AMA3 and CR2-AMF1 spores were confirmed for their generic identities using 18S rRNA gene sequence data (Table 4 and Figure 9). CM2-AMA3 spores related closely to *Acaulospora spinosa* Att165-9 with 98.92% gene sequence similarity, while CR2-AMF1 spores exhibited 99.86% similarity to *Funneliformis mosseae* BEG12.

### 2.4. Roles of PGPR and AM Fungi in the Development and Growth of Physic Nut and Sacha Inchi

**Figure 10:**
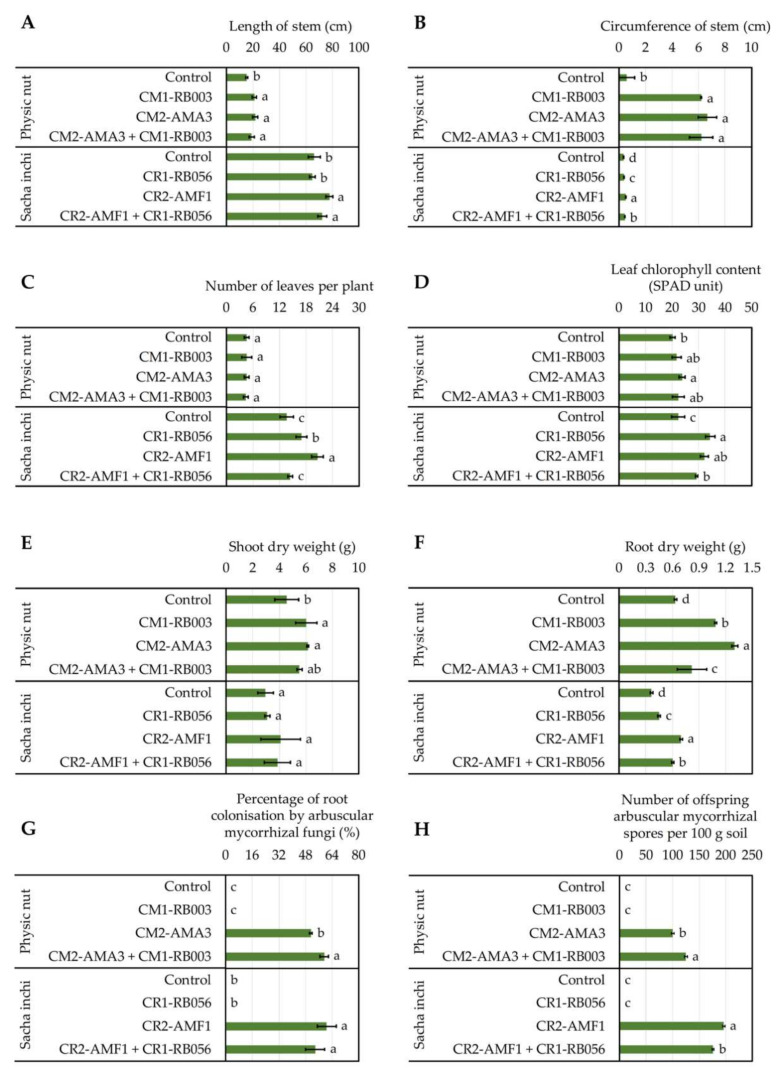
Impacts of rhizosphere microbes on the development and growth of physic nut and sacha inchi (**A**–**F**) together with the proliferation of arbuscular mycorrhizal (AM) fungi (**G**,**H**) assessed in pot experiments. The treatments comprised of uninoculated controls and those inoculated with rhizobacteria (*Ensifer* sp. CM1-RB003 or *Pantoea* sp. CR1-RB056), AM fungi (*Acaulospora* sp. CM2-AMA3 or *Funneliformis* sp. CR2-AMF1), or mixtures of both microbes. The experiments were done in five replicates. Lower-case letters refer to the statistical difference of means derived from any treatments per plant species, compared by one-way ANOVA. The statistical results are as follows. Stem lengths (**A**) of physic nut (*F*_(3, 16)_ = 15.10, *p* ≤ 0.0005) and sacha inchi (*F*_(3, 16)_ = 16.20, *p* ≤ 0.0005). Stem circumferences (**B**) of physic nut (*F*_(3, 16)_ = 100.30, *p* ≤ 0.0005) and sacha inchi (*F*_(3, 16)_ = 152.40, *p* ≤ 0.0005). Leaf numbers per plant (**C**) of physic nut (*F*_(3, 16)_ = 0.091, *p* ≤ 0.964) and sacha inchi (*F*_(3, 16)_ = 33.72, *p* ≤ 0.0005). Leaf chlorophyll contents (**D**) of physic nut (*F*_(3, 16)_ = 4.33, *p* ≤ 0.02) and sacha inchi (*F*_(3, 16)_ = 47.56, *p* ≤ 0.0005). Shoot dry weights (**E**) of physic nut (*F*_(3, 16)_ = 6.94, *p* ≤ 0.003) and sacha inchi (*F*_(3, 16)_ = 1.74, *p* ≤ 0.199). Root dry weights (**F**) of physic nut (*F*_(3, 16)_ = 60.12, *p* ≤ 0.0005) and sacha inchi (*F*_(3, 16)_ = 577.49, *p* ≤ 0.0005). Percentages of root colonization by AM fungi (**G**) for physic nut (*F*_(3, 16)_ = 2853.85, *p* ≤ 0.0005) and sacha inchi (*F*_(3, 16)_ = 342.42, *p* ≤ 0.0005). Numbers of offspring AM spores produced in rhizosphere soils (**H**) of physic nut (*F*_(3, 16)_ = 6654.59, *p* ≤ 0.0005) and sacha inchi (*F*_(3, 16)_ = 26,070.47, *p* ≤ 0.0005).

The best IAA-forming rhizobacteria (i.e., *Ensifer* sp. CM1-RB003 from physic nut and *Pantoea* sp. CR1-RB056 from sacha inchi) and the most prolific AM fungi (i.e., *Acaulospora* sp. CM2-AMA3 from physic nut and *Funneliformis* sp. CR2-AMF1 from sacha inchi) were tested either individually or in consortium for their plant growth-promoting potentials in pot experiments (Figure 10, Figure S3, and Figure S4). The lengths and the circumferences of physic nut stems were significantly longer in the plants treated with microorganisms compared to non-inoculated controls (Figure 10A,B and Figure S3). On the other hand, in sacha inchi, the best values were achieved by plants treated with AM fungi (Figure 10). Numbers of leaves per plant and leaf chlorophyll contents for physic nut were not affected by any treatments (Figure 10C,D). However, in sacha inchi, rhizobacteria, AM fungi, and consortium treatments increased the numbers of leaves, and rhizobacteria and AM fungi treatments increased the leaf chlorophyll contents (Figure 10C,D) compared to the non-inoculated control (Figure 10C). Shoot and root dry weights of physic nut were increased by all microbial treatments (Figure 10D,E). A similar trend was observed also for the root dry weights of sacha inchi (Figure 10E).

The status of AM proliferation in pot experiments was also assessed (Figure 10G,H). No AM fungi were detected in the non-inoculated control. Interestingly, in physic nut (Figure 10G,H), the percentages of root colonization by AM fungi and the numbers of offspring AM spores were higher in plants treated with the consortium in comparison with the plant treated with only the AM fungi.

## 3. Discussion

To our knowledge, little is known about the rhizosphere microbial community of oil crops. Some studies reported different bacterial counts in physic nut rhizospheres established in diverse soils, e.g., wasteland [49] and tsunami-perturbed [54] soils. With these studies, it seems that soil fertility and physicochemical properties are the critical factors influencing the bacterial abundance in physic nut rhizospheres. However, neither plant species, geographical locations, nor soil physicochemical properties significantly affected the rhizobacterial abundance of either oil crop (physic nut and sacha inchi) assessed in our study. With our culture-dependent assessments, ~10^6^ rhizobacteria dwelling on surfaces of 50 g roots of both oil crops were recorded. This rhizobacterial number is lower than a typical number (10^8^–10^12^ cells) of bacteria per 1 g of rhizosphere soils [65], which can be a result of the differences in the sources for bacterial isolation and the isolation techniques. In our study, we only quantified the rhizobacteria residing in the proximity of plant roots (root surfaces), not in the bulk rhizosphere soils where the synergistic interactions between microbes and plant roots are yet vague. Furthermore, we used a low-nutrient medium without any nitrogen source for bacterial isolation, which was more optimal for a certain group of bacteria to grow (e.g., nitrogen-fixing bacteria) [66]. Besides, it is hard to compare the bacterial richness using culture-based techniques because many viable but unculturable bacteria in nature cannot grow under laboratory conditions. Hitherto, there is only one available study assessing the rhizosphere microbial abundance and diversity of sacha inchi grown in different land-use conversion soils, using a set of molecular approaches [63]. Although the authors reported a high range of bacterial copies (10^10^–10^11^) detected in this plant’s rhizospheres established in the diverse soils tested, these copy numbers are a sum of both viable and non-viable bacterial counts. The AM fungal status of oil crop rhizospheres is also rarely known. A study explored the AM fungal abundance of physic nut grown in various areas in Thailand and reported that over 50 AM spores found in 100 g rhizosphere soil referred to a high degree of AM fungal abundance [56]. In our study, rhizospheres of physic nut and sacha inchi hold abundant AM spores in a range of 395–560 spores per 100 g soil. The soil phosphorus level exhibited an apparent effect on the AM fungal abundance of both oil crops, regardless of plant species and geographical locations (Table 1 and Table 5). Over 500 AM spores were detected in phosphorus-rich (>200 mg kg^−1^) soils at cultivation sites CM2 and NR2, while the lowest AM spore count was found in the soil with a low level of phosphorus (<150 mg kg^−1^). A study unveiled that the growth of physic nut was highly dependent upon the support by AM fungi, especially at a low level of soil phosphorus (<50 mg kg^−1^) [10]. However, the authors did not report the abundance of and the ability to colonize plant roots by the AM fungi tested. 

It is conceivable that commensal rhizobacteria are responsible for the optimized growth and development of their mutual plants. Some studies proved that many *Enterobacter* isolates resided in the rhizospheres and roots of physic nut and promoted the growth of this plant [51,52,53]. However, this bacterial genus was not identified in our study. As the environmental conditions of rhizosphere samples and the selection criteria of rhizobacteria for identification were different, we could not conclude that *Enterobacter* is not among PGPR of physic nut assessed in our study. The ability to produce a high level of IAA was the criterion to determine the best performance of our isolated rhizobacteria. These IAA-forming bacteria from physic nut rhizospheres were the members of two bacterial classes, *Alphaproteobacteria* (*Ensifer* sp. and *Agrobacterium* sp.) and *Gammaproteobacteria* (*Raoultella* sp. and *Pseudomonas* spp.). Jha et al. [49] reported that *Pseudomonas* was the most abundant bacterial genus found in rhizospheres of physic nut grown in wasteland soil. In contrary to physic nut rhizospheres, sacha inchi rhizospheres housed more variety of IAA-forming rhizobacteria that belonged to three classes, *Gammaproteobacteria* (*Pantoea* sp.), *Actinobacteria* (*Microbacterium* sp.), and *Betaproteobacteria* (*Burkholderia* sp.). Wang et al. [63] revealed that *Gammaproteobacteria* and *Actinobacteria* were the bacterial members of sacha inchi rhizospheres. The difference in rhizobacterial diversity seemed to be a result of different mutual plant species. Different plant species produce dissimilar root exudates, which are the key factors shaping different community structures of commensal rhizosphere microbes [20,21,22,23,24]. However, for oil crops, further investigations using recent omics tools in molecular biology would provide in-depth elucidation of microbial diversity dwelling in their rhizospheres.

After classifying AM spores into their morphological groups (Table 5) and testing their ability to colonize plant roots and produce offspring spores (Figure 7), not every AM fungal group could proliferate. For examples, 225 NR1-AMA6 spores were found per 100 g rhizosphere soil of physic nut grown at site NR1 (Table 5), which was relatively abundant, but they could not propagate during root colonization tests. This loss in propagation may be a result of the changes in abiotic and biotic components of the matrix used to grow plants in our study, as a sterilized soil and sand mixture was used. The most prolific AM fungi that offered high percentages of root colonization and produced high numbers of offspring spores were *Acaulospora* sp. CM2-AMA3 and *Funneliformis* sp. CR2-AMF1 derived from physic nut and sacha inchi, respectively. Based on our AM fungal diversity study, only two AM genera (*Acaulospora* and *Glomus*) were found in physic nut rhizospheres. In contrast, more diverse AM genera (*Acaulospora*, *Claroideoglomus*, *Glomus*, and *Funneliformis*) were detected in sacha inchi rhizospheres. *Acaulospora* and *Glomus* seemed to be typical AM fungi that live in soil associated with physic nut, supported by a study unveiling that rhizosphere soils of this oil crop grown at different locations in Thailand housed diverse AM fungi, including *Acaulospora*, *Entrophospora*, *Gigaspora*, *Glomus*, and *Scutellospora* [55]. For sacha inchi, our study is the first to elucidate the AM fungal diversity of this oil crop. However, a set of previous studies reported diverse novel AM taxa from sacha inchi rhizospheres, including *Acaulospora aspera*, *Funneliglomus sanmartinensis,* and *Microkamienskia peruviana* [60,61,62]. With these studies and our findings, *Acaulospora* would be the predominant AM fungal genus found in sacha inchi rhizospheres. Although a piece of molecular evidence proved that sacha inchi rhizosphere soils hold 10^10^–10^11^ fungal copy numbers [63], none of AM fungi were identified in such study. This identification limit of AM fungi may be due to the absence of AM fungi in soils or the inadequate DNA extraction as AM spores have strong multilayer walls that need a physical force to breakdown for accessing their nucleic materials. The difference in AM fungal diversity depended highly on the oil crop species. However, we also observed that potassium levels of all physic nut rhizosphere soils were significantly higher (>318 mg kg^−1^) compared to those of sacha inchi (<278 mg kg^−1^). To our knowledge, the impacts of soil potassium level on the abundance and diversity of AM fungi are unknown. This soil chemical signature may play an important role in shaping the community structure of AM fungi; hence, further investigations are yet needed.

Some studies demonstrated the roles of PGPR and/or AM fungi in enhancing the development, growth and/or yield of physic nut [10,50,51,52,53,57,58,59] and sacha inchi [64] grown in diverse soils. Similarly, our pot experiments using either PGPR or AM fungi derived from both oil crops exhibited the enhanced development and growth of these plants in most treatments, compared to the non-inoculated controls. To our knowledge, the plant growth-promoting roles of sacha inchi rhizobacteria are yet unknown; we reported in this study, for the first time, that *Pantoea* sp. CR1-RB056 is among the promising beneficial rhizobacteria of sacha inchi. This rhizobacterium can increase stem width, leaf number, leaf chlorophyll content, and root dry weight of sacha inchi grown in the sterilized matrix. For plant growth-promoting assessments of AM fungi, the percentage of root colonization by AM fungi and the number of their produced offspring spores in the rhizosphere soil were the crucial parameters, indicating their proliferation and whether they had synergistic interactions with other with added rhizobacteria or not. It was evident in the pot experiments with sacha inchi that the proliferation of the AM fungus *Funneliformis* sp. CR2-AMF1 was diminished in the presence of the rhizobacterium *Pantoea* sp. CR1-RB056. This adverse interaction was supported by the significant decrease in produced offspring AM spores in the presence of the rhizobacterium, which also resulted in reduced plant growth-promoting activities (Figure 10). Cautiously, the adverse interaction between AM fungi and rhizobacteria should be avoided, while either applying individual microbe or searching for other compatible matches could be solutions. In contrast to the pot experiments with physic nut, the rhizobacterium *Ensifer* sp. CM1-RB003 could promote the proliferation of the AM fungus *Acaulospora* sp. A similar finding was observed with the supportive interactions between AM fungal consortia and *Azotobacter chroococcum*, and both rhizosphere partners could enhance the biomass yield of physic nut [59].

## 4. Materials and Methods 

### 4.1. Sampling Sites and Sample Collection 

Physic nut-related samples were collected from sites CM1, CM2, NR1, and NR2. Sites CM1 and CM2 are in the experimental farm area of Mae Jo University, Chiang Mai, Thailand, while sites NR1 and NR2 are in the organic farm area of Suranaree University of Technology, Nakhon Ratchasima, Thailand. An approximate amount (~1.5 kg) of rhizosphere soil-containing plant roots was sampled at 8 cm beyond the trunk flare and a 10-cm depth from the ground and kept in a plastic bag. At the same site, four samples (~1.5 kg each) were collected randomly from four plants. The samples were preserved in an icebox before transporting to the laboratory. The same sampling procedures were conducted for the specimens derived from sacha inchi growing at sites CR1 and CR2 in Chiang Rai, Thailand. The geographical location, host plant, and age, together with sampling time, are listed in Table 1. All four samples derived from each site were pooled together and homogenized well before use.

### 4.2. Soil Physicochemical Analyses 

Plant roots were separated from all samples. The root-free soil samples were air-dried before used for physicochemical analyses provided by an internal service of the Department of Soil Science, Faculty of Agriculture, Chiang Mai University, Chiang Mai, Thailand. Briefly, percentages of total nitrogen and organic matter were quantified using Kjeldahl’s and Walkley-Black chromic acid wet oxidation methods, respectively. A soil:distilled water ratio of 1:1 (*w*/*v*) was prepared for measuring soil pH with a pH meter. Available phosphorus and exchangeable potassium were determined by Jackson’s method. The soil physicochemical properties at each sampling site are listed in Table 1.

### 4.3. Isolation of Rhizosphere Microbes

Rhizobacteria were isolated from the surface of plant roots. Fifty grams of plant roots was suspended in 10 mL 0.85% (*w*/*v*) sterile normal saline and vortexed for 5 min, four times. The suspension was 10-fold serially diluted using sterile normal saline to reach the dilution factor of 10^−6^. To selectively isolate nitrogen-fixing rhizobacteria, the selected dilution (10^−3^–10^−6^) was spread over a modified Burk’s nitrogen-free agar medium (compositions per 1 L: 10 g glucose, 0.41 g KH_2_PO_4_, 0.52 g K_2_HPO_4_, 0.05 g Na_2_SO_4_, 0.2 g CaCl_2_, 0.1 g MgSO_4_·H_2_O, 0.005 g FeSO_4_·7H_2_O, 0.0025 g Na_2_MO_4_·2H_2_O, and 15 g agar) [66] and incubated at 30 °C for 3–7 days. After incubation, total bacterial counts were recorded, and 15% of differentially appeared bacterial colonies per site were randomly picked and sub-cultured on the same agar medium to confirm their viability until becoming axenic cultures. The cultures were maintained in 15% (*v*/*v*) glycerol stock at −20 °C before further use.

Twenty grams of soil sample was assessed for the initial AM spore counts, following modified wet sieving and the sucrose centrifugation method described by Chaiyasen et al. [36]. Briefly, soil suspended with tap water was passed through a series of sieves with different mesh sizes (250, 106, and 45 µm). AM spores were collected on filter paper (Whatman No. 1) and counted under a stereomicroscope (Olympus SZ40, Olympus Optical Co., Ltd., Tokyo, Japan). The assessment was conducted five times (a total of 100 g soil used). The number of total spores collected per time was recorded, and spores derived from the same sampling site were pooled together for further studies.

### 4.4. Screening of Plant Growth-Promoting Activity

Plant growth-promoting activities (i.e., productions of indole-3-acetic acid (IAA) and siderophore, nitrogen fixation, and phosphate solubilization) of every rhizobacterial isolate were evaluated. The ability to form IAA-like molecules was primarily screened following a modified protocol described by Nakaew et al. [67]. Briefly, cell-free culture broth of a 3-day-old rhizobacterial isolate, grown previously in 5 mL nutrient broth (NB) (HiMedia, Mumbai, India) supplemented with 2% (*w*/*v*) tryptophan, was mixed with Salkowski’s reagent (composed of 2 mL 0.5M FeCl_3_, 49 mL water, and 49 mL 70% HClO_4_). The mixture was homogenized and incubated in the dark for 30 min before measuring its absorbance at a 530-nm wavelength using a spectrophotometer (Genesys 20, Thermo Fisher Scientific Inc., Waltham, MA, USA). A pink–red color corresponds to the positive production of IAA-like molecules. NB without rhizobacterial inoculum was used as a control. The quantity of produced IAA-like molecules was estimated in comparison with a standard curve generated by different concentrations of IAA.

The top three rhizobacterial isolates per each studied site that produced the highest level of IAA-like molecules were confirmed for their ability to produce IAA by a HPLC analysis. Briefly, a HPLC Shimadzu LC10-ADVP (Shimadzu Co., Kyoto, Japan) with Restek Ultra C18 (5.0 mm × 15 mm, 5 μm), was conducted by an internal service of the Agricultural Technology Services Center, Faculty of Agriculture, Chiang Mai University, Chiang Mai, Thailand. Here, 65% of 2.5% acetic acid:acetonitrile, pH 3.6, adjusted with KOH, was used as mobile phase A, and 35% of 80% acetonitrile in deionized water served as mobile phase B. The column temperature was 25 °C, with a flow rate of 0.85 mL min^−1^, and a diode-array detector was set at 280 nm. Then, 20 mL of sample was injected within 15 min of run time [68].

The ability to produce siderophore was tested following a modified protocol described by Rangjaroen et al. [69]. Briefly, each rhizobacterial isolate was grown on chrome azurol S agar medium at ambient temperature (~25 °C) for 48 h. Diameters of yellow–orange zones appeared around bacterial colonies corresponding to the levels of siderophore produced by tested rhizobacteria.

The ability to fix atmospheric nitrogen was determined with a modified acetylene reduction assay described by Prakamhang et al. [70]. Briefly, the nitrogenase activity of each rhizobacterial isolate was assayed after its growth in 5 mL Burk’s nitrogen-free broth [65] at ambient temperature (~25 °C) for 48 h on a rotatory shaker at 110 rpm. Then, the air in the culture tube’s headspace was replaced with acetylene gas at a final concentration of 10% (*v*/*v*) and incubated at ambient temperature (~25 °C) for 24 h. The presence of nitrogenase corresponds to the ethylene gas produced in the headspace, which was quantified by gas chromatography equipped with a flame ionization detector and a PE-Alumina column, 50 m × 0.32 mm × 0.25 µm (PerkinElmer, Bellefonte, PA, USA). The specific nitrogenase activity was reported as nmol h^−1^ per mg cell protein, while the cell protein was measured following a protocol described by Desvaux et al. [71].

The ability to solubilize phosphate was examined following the method described by Chaiharn and Lumyong [29]. Briefly, each rhizobacterial isolate was cultivated on Pikovskaya’s agar medium (compositions per L: 10 g glucose, 0.2 g KCl, 0.5 g (NH_4_)_2_SO_4_, 0.2 g NaCl, 0.1 g MgSO_4_·7H_2_O, 0.002 g MnSO_4_·H_2_O, 0.002 g FeSO_4_·7H_2_O, 5 g Ca_3_(PO_4_)_2_, and 15 g agar) at ambient temperature (~25 °C) for 7 days. Diameters of halo zones that appeared around the bacterial colonies correspond to the levels of phosphate solubilized by the tested rhizobacteria.

### 4.5. Morphological Categorisation and Viability Confirmation of Isolated AM Fungi

Morphological characteristics (e.g., color, spore surface, structures of spore wall, subtending hypha, etc.) of isolated AM spores were used to categorize them into different groups. AM spores were mounted on microscopic slides with polyvinyl alcohol-lactic acid-glycerol (PVLG) or a mixture of PVLG and Melzer’s reagent at a ratio of 1:1 (*v*/*v*). The prepared slides were observed under a light microscope (Olympus CH30, Olympus Optical Co., Ltd., Tokyo, Japan). The identification and classification were performed using the spore descriptions available at http://fungi.invam.wvu.edu/the-fungi/species-descriptions.html. The fertility of representative spores from each morphological group was assessed under the microscope, and the fertile ones were subjected to the spore propagation test with their host plant.

Briefly, surfaces of physic nut and sacha inchi seeds were sterilized, using a modified protocol described elsewhere in [56], by soaking seeds in 7% (*v*/*v*) NaOCl for 5 min and washed five times with sterile distilled water. The prepared seeds were sown on a sterilized matrix containing 1:1 (*v*/*v*) soil:sand (autoclaved twice at 121 °C, 15 psi for 15 min) to allow germination for 15 days. A germinated plant seedling was transferred into a pot containing 2000 cm^3^ of the same matrix mentioned, and 50 AM spores were inoculated at the root zone. All experiments were conducted following a completely randomized design (CRD) with five replicates per spore group, under greenhouse conditions with 12 h light/dark cycle for 3 months. The modified Hoagland’s nutrient solution was applied to each pot once per week [36]. At the end of the experiments, rhizosphere soil was sampled from each pot for counting offspring AM spores, following the protocol described before. Plant roots were also collected to confirm the root colonization by AM fungi. Root segments were observed under a light microscope (Olympus BH2, Olympus Optical Co., Ltd., Tokyo, Japan) equipped with an Olympus OM-D E-M10 II digital camera, following the procedures described elsewhere in [36,72]. The data were reported as the percentage of root colonization by AM fungi.

### 4.6. Genotypic Identification and Phylogenetic Analysis of Selected Rhizosphere Microbes

Some rhizobacterial isolates were selected based on their promising IAA-forming activity and identified using 16S rRNA gene sequencing. The bacteria were grown overnight in NB at ambient temperature (~25 °C) with shaking at 150 rpm. Bacterial cells were collected and used for DNA extraction with Quick-DNA^TM^ Universal Kit (Zymo Research, Irvine, CA, USA), following the manufacturer’s instruction. The 16S rRNA gene was amplified using primers 27F (5′-AGAGTTTGATCCTGGCTCAG-3′) and 1492R (5′-GGTTACCTTGTTA CGACTT-3′) [73]. Polymerase chain reaction (PCR) mixture contained 10 µL of 2 × PCR Master Mix Solution (i-*Taq*^TM^) (Thermo Fisher Scientific Inc., Waltham, MA, USA), 1 µL of each primer (10 pmol µL^−1^), 2 µL of DNA template, and nuclease-free water to adjust the volume up to 20 µL. PCR was conducted in a Bio-Rad PCR Thermal Cycler with a thermal program: 94 °C for 5 min, 30 cycles of 95 °C for 30 s, 55 °C for 30 s and 72 °C for 1 min, and 72 °C for 7 min. 

Some AM fungi, selected based on root-colonizing ability, were identified using 18S rRNA gene sequencing. The surface of an AM spore was sterilized using 2% (*w*/*v*) chloramine T, 0.1% (*w*/*v*) streptomycin sulphate, and 0.05% (*w*/*v*) gentamicin sulphate [36]. The prepared spore was physically broken in a 0.2-mL PCR tube containing 5 µL of nuclease-free water, using a pipette tip, and the suspension served as a DNA template. A nested PCR targeting the AML1–AML2 region in the 18S rRNA gene [74] was conducted for the first step with primers NS1 5′-GTAGTCATATGCTTGTCTC-3′ and NS4 5′-CTTCCGTCAATTCCTTTAAG-3′ [75] and for the second step with primers AML1 5′-ATCAACTTTCGATGGTAGGAT-3′ and AML2 5′-GAACCCAAACACTTTGGT-3′ [76]. All PCR mixtures (20 µL) contained 10 µL of 2 × PCR Master Mix Solution (i-*Taq*^TM^), 1–2 µL of DNA template, 1 µL of each primer (10 pmol µL^−1^), and nuclease-free water for the volume adjustment. The thermal program for the first amplification was 95 °C for 3 min, 40 cycles of 94 °C for 30 s, 55 °C for 40 s and 72 °C for 1 min, and 72 °C for 5 min, and that for the second amplification was 94 °C for 3 min, 35 cycles of 94 °C for 1 min, 50 °C for 1 min and 72 °C for 1 min, and 72 °C for 10 min. 

PCR products were sequenced using their corresponding PCR primers (27F and 1492R for 16S rRNA gene sequences and AML1 and AML2 for 18S rRNA gene sequences) by an external service provided by 1st Base (Selangor, Malaysia). The nucleotide sequences were edited and identified using publicly available databases in GenBank via BLASTn (https://blast.ncbi.nlm.nih.gov/Blast.cgi) and EzBioCloud (https://www.ezbiocloud.net/) for bacterial sequences or MycoBank (https://www.mycobank.org/) for AM sequences. All relevant sequences were collected and used for multiple sequence alignments with the MUSCLE algorithm available in MEGA 7 software (https://www.megasoftware.net/), where either neighbor-joining or maximum likelihood phylogenetic trees were constructed with the Tamura-Nei model and bootstrap values based on 1000 replications. Pairwise-compared sequence similarities were computed with PHYDIT program version 1.0 (http://plaza.snu.ac.kr/~jchun/jphydit/index.php).

### 4.7. Pot Experiments to Access Plant Growth-Promoting Potentials of Selected Rhizosphere Microbes

The rhizobacteria with the highest IAA production confirmed by HPLC and the AM fungi with the highest root colonization values were used for pot experiments. The overnight culture of each bacterium in NB was centrifuged at 6000× *g* for 10 min to collect its biomass. The biomass was adjusted with sterile distilled water to the optical density at a 600-nm wavelength of 1.2, which corresponded to 10^8^ CFUs mL^−1^. The propagation and collection of AM spores were conducted as described elsewhere. Seedlings of physic nut and sacha inchi were prepared after seed surface sterilization and cultivation in sterilized matrix for 4 weeks, as mentioned before.

Pot experiments were carried out considering four different treatments, including (1) non-inoculated seedlings (control) and seedlings inoculated with (2) rhizobacterial inoculum, (3) AM inoculum, or (4) both rhizobacterial and AM inocula. Each seedling was planted in the center of a pot (20-cm Ø, 18-cm height) containing the same matrix used for the seedling preparation at 2000 cm^3^. For treatments 2 and 4, 30 mL of the prepared bacterial suspension was added at the root zone, while for the control and treatment 3, 30 mL of sterile distilled water was added to equilibrate the matrix moisture content. For treatments 3 and 4, 100 AM spores were inoculated into the root zone. The experiments were conducted following CRD with 5 pots per treatment under greenhouse conditions with 12-h light/dark cycle for 3 months. Each pot was watered with 100 mL tap water once a day and supplemented with 50 mL Hoagland’s nutrient solution once a week [36].

Plant growth indexes, i.e., length of stem, circumference of stem, number of leaves per plant, leaf chlorophyll content, shoot dry weight, and root dry weight, together with the proliferation status of AM fungi (i.e., number of offspring AM spores produced and percentage of plant root colonization), were measured and recorded at the end of the experiments. The leaf chlorophyll content was measured using a chlorophyll meter (SPAD-502Plus, Konica Minolta Sensing Inc., Osaka, Japan) and reported in the Soil Plant Analysis Development (SPAD) unit. All plants were uprooted and divided into shoots and roots. Rhizosphere soil was collected for determining the offspring AM spores in 100 g soil, following the protocol mentioned before. Roots were washed and used to assess the percentage of root colonization by AM fungi as described previously. Shoot and root dry weights were measured after washing plant materials through running tap water and drying in a hot air oven at 70 °C for 7 days.

### 4.8. Statistical Analysis

All means ± standard deviations (SDs) were statistically compared using one-way analysis of variance (ANOVA) with Tukey’s post hoc test, available in SPSS Statistics software version 26 (IBM Corporation, Somers, NY, USA). The significant values tested at *p* = 0.05 and/or *p* = 0.01 were reported with *F*-distribution values and their degrees of freedom.

## 5. Conclusions

Rhizospheres of oil crops, physic nut and sacha inchi house diverse PGPR and AM fungi, and these rhizosphere microbes play significant roles in the development and growth of both oil crops. With the culture-dependent approach, there was no influence regarding different oil crop species, geographical locations, and soil physicochemical properties on the abundance of rhizobacteria. For AM fungal abundance, it is highly dependent upon the soil phosphorus levels. The difference in oil crop species is a key factor shaping the diversity of their rhizosphere microbes, as a higher variety of microbial taxa were found in sacha inchi rhizospheres than in physic nut rhizospheres. However, soil potassium levels seem to play some roles in AM fungal diversity, which requires further investigations to fill this knowledge gap. Plant growth-promoting assessments of selected rhizosphere microbes in the pot experiments confirm that either individuals or consortia of these microbes can maintain or enhance the growth and development of both oil crops, and the levels of enhancement rely on the microbial species chosen, particularly when applying in the form of microbial consortia. The beneficial rhizosphere microbes obtained from this study can be applied further as biostimulants to optimize oil crop production.

## Figures and Tables

**Table 1:** Geographical locations, host plants, and soil physicochemical properties of sampling sites.

Soil Characteristic	Sampling Site					
	CM1	CM2	NR1	NR2	CR1	CR2
Geographical location (latitude–longitude)	Chiang Mai (18°53′41.6′′ N–99°00′54.3′′ E)	Chiang Mai (18°53′48.9′′ N–99°01′06.2′′ E)	Nakhon Ratchasima (14°52′53.4′′ N–102°01′14.4′′ E)	Nakhon Ratchasima (14°87′20.27′′ N–102°02′57.44′′ E)	Chiang Rai (20°02′01.1′′ N–99°51′11.9′′ E)	Chiang Rai (20°02′03.5′′ N–99°51′08.6′′ E)
Host plant (age)	Physic nut (~7 years)	Physic nut (~7 years)	Physic nut (~5 years)	Physic nut (~5 years)	Sacha inchi (~5 years)	Sacha inchi (~5 years)
Sampling time	August 2014	August 2014	November 2014	November 2014	January 2016	January 2016
Total nitrogen (%)	0.19 ± 0.01ab	0.22 ± 0.01a	0.16 ± 0.02bc	0.18 ± 0.02b	0.13 ± 0.02c	0.16 ± 0.01bc
Available phosphorus (mg kg^−1^)	137.49 ± 0.77b	204.01 ± 7.26a	157.81 ± 2.88b	204.53 ± 0.91a	143.02 ± 1.36b	150.24 ± 0.98b
Exchangeable potassium (mg kg^−1^)	383.29 ± 2.17b	318.26 ± 1.75d	452.35 ± 1.64a	329.98 ± 1.13c	256.29 ± 1.12e	278.34 ± 0.76e
Organic matter (%)	3.30 ± 0.02c	4.80 ± 0.02a	3.35 ± 0.09c	3.90 ± 0.03b	3.50 ± 0.07c	3.78 ± 0.06c
pH in 1:1 (*w*/*v*) soil: distilled water	7.78 ± 0.06b	7.90 ± 0.06a	7.92 ± 0.03a	7.31 ± 0.03c	7.02 ± 0.03d	7.02 ± 0.03d

Numerical data are means ± SDs derived from three technical replicates. Different lower-case letters indicate the statistical difference of means between sites analyzed by one-way ANOVA, i.e., total nitrogen (*F*_5, 12)_ = 15.88, *p* ≤ 0.0005), available phosphorus (*F*_(5, 12)_ = 15.25, *p* ≤ 0.0005), exchangeable potassium (*F*_(5, 12)_ = 7269.82, *p* ≤ 0.0005), organic matter (*F*_(5, 12)_ = 78.58, *p* ≤ 0.0005), and pH (*F*_(5, 12)_ = 328.93, *p* ≤ 0.0005).

**Table 2:** Coding and numbers of rhizobacteria derived from physic nut and sacha inchi.

Cultivation Site *^a^*	Host Plant	Total Appeared Rhizobacterial Colonies/50 g Roots	Number of Selected Rhizobacterial Isolates/50 g Roots	Codes of Selected Rhizobacterial Isolates
CM1	Physic nut	336	51	CM1-RB001–CM1-RB051
CM2	Physic nut	417	63	CM2-RB001–CM2-RB063
NR1	Physic nut	514	77	NR1-RB001–NR1-RB077
NR2	Physic nut	265	40	NR2-RB001–NR2-RB040
CR1	Sacha inchi	627	94	CR1-RB001–CR1-RB094
CR2	Sacha inchi	470	71	CR2-RB001–CR2-RB071
Total		2629	396	

*^a^* Information about cultivation sites and host plants is available in Table 1.

**Table 3:** Plant growth-promoting activities of selected rhizobacteria.

Rhizobacterial Isolate *^a^*	Indole-3-Acetic Acid Production (µg mL^−1^) *^b^*		Nitrogen Fixation (Nitrogenase Activity, nmol h^−1^ per mg Cell Protein)	Siderophore Production (Producing Zone, mm ø)	Phosphate Solubilization (Solubilizing Zone, mm ø)
	Colorimetry	HPLC			
CM1-RB003	196.36 ± 0.88	30.67 ± 0.54	91.99 ± 1.99	17.67 ± 1.53	-
CM1-RB020	39.55 ± 1.33	9.40 ± 0.27	-	7.00 ± 0.00	-
CM2-RB021	44.81 ± 7.62	3.96 ± 0.24	83.17 ± 2.72	-	6.67 ± 1.15
NR1-RB026	133.14 ± 4.01	14.03 ± 1.86	-	-	-
NR2-RB004	32.36 ± 4.67	2.10 ± 0.47	-	11.33 ± 0.58	-
CR1-RB056	66.09 ± 17.80	24.73 ± 7.90	77.59 ± 2.29	-	-
CR2-RB043	103.25 ± 10.14	23.10 ± 5.23	-	-	-
CR2-RB046	86.44 ± 14.53	15.51 ± 3.35	-	-	-

*^a^* The origins of these bacteria are available in Table 1, Table 2 and Table 4. All measuring methods to retrieve the plant growth-promoting activities are available elsewhere in the text. *^b^* The ability to form indole-3-acetic acid was assessed with either the colorimetric method or high-performance liquid chromatography (HPLC). All reported values are means ± standard deviations derived from triplicate measurements. All results marked with (-) refer to ‘no activity’.

**Table 4:** Rhizosphere microbes of physic nut and sacha inchi and their related phylogenetic species.

Microbial Isolate *^a^* (GenBank Accession Number)	Closest Phylogenetic Species (GenBank Accession Number)	Pairwise-Compared Sequence Similarities (%)
Rhizobacteria		
CM1-RB003 (MT860436)	*Ensifer sesbaniae* CCBAU 65729^T^ (JF834143)	99.78
CM1-RB020 (MT860437)	*Agrobacterium radiobacter* IAM 12048^T^ (AB247615) *Agrobacterium tumefaciens* NCPPB 2437^T^ (AB247617)	98.99
CM2-RB021 (MT860438)	*Raoultella planticola* ATCC 33531^T^ (FJ971885)	98.92
NR1-RB026 (MT860439)	*Pseudomonas lactis* DSM 29167^T^ (KP756923)	97.31
NR2-RB004 (MT860440)	*Pseudomonas mosselii* CIP 105259^T^ (AF072688)	99.71
CR1-RB056 (MT860441)	*Pantoea wallisii* LMG 26277^T^ (FJ295057)	99.16
CR2-RB043 (MT860442)	*Microbacterium mangrovi* MUSC 115^T^ (KF028598)	98.65
CR2-RB046 (MT860443)	*Burkholderia contaminans* LMG 23361^T^ (JX986975) *Burkholderia paludis* MSh1^T^ (KT159931)	99.93
Arbuscular mycorrhizal fungi		
CM2-AMA3 (MT860453)	*Acaulospora spinosa* Att165-9^T^ (FR750204)	98.92
CR2-AMF1 (MT860454)	*Funneliformis mosseae* BEG12^T^ (JX461236)	99.86

*^a^* The origins of these bacteria are available in Table 1, Table 2 and Table 4.

**Table 5:** Codes, numbers, and morphological groups of arbuscular mycorrhizal fungi derived from physic nut and sacha inchi rhizosphere soils.

Arbuscular Mycorrhizal Fungi (Morphological Group)	Spore Codes at Different Cultivation Sites *^a^* (Number of Spores Detected per 100 g Soil)					
	CM1	CM2	NR1	NR2	CR1	CR2
*Acaulospora* sp. (A1)	CM1-AMA1 (120)	CM2-AMA1 (90)	NR1-AMA1 (180)	-	-	-
*Acaulospora* sp. (A2)	-	CM2-AMA2 (60)	-	NR2-AMA2 (60)	-	CR2-AMA2 (40)
*Acaulospora* sp. (A3)	CM1-AMA3 (145)	CM2-AMA3 (190)	-	NR2-AMA3 (225)	-	-
*Acaulospora* sp. (A4)	CM1-AMA4 (25)	CM2-AMA4 (30)	-	-	-	-
*Acaulospora* sp. (A5)	CM1-AMA5 (40)	-	-	-	-	-
*Acaulospora* sp. (A6)	-	-	NR1-AMA6 (255)	NR2-AMA6 (125)	-	-
*Acaulospora* sp. (A7)	CM1-AMA7 (65)	CM2-AMA7 (90)	-	NR2-AMA7 (110)	-	-
*Acaulospora* sp. (A8)	-	-	-	-	CR1-AMA8 (115)	CR2-AMA8 (110)
*Acaulospora* sp. (A9)	-	-	-	-	-	CR2-AMA9 (50)
*Claroideoglomus* sp. (C1)	-	-	-	-	CR1-AMC1 (90)	-
*Glomus* sp. (G1)	CM1-AMG1 (85)	CM2-AMG1 (100)	-	-	-	-
*Glomus* sp. (G2)	-	-	-	-	CR1-AMG2 (60)	CR2-AMG2 (50)
*Funneliformis* sp. (F1)	-	-	-	-	CR1-AMF1 (130)	CR2-AMF1 (170)
Total number of spores detected per 100 g soil per site	480	560	435	520	395	420

*^a^* Information about the cultivation sites and host plants is available in Table 1. All results marked with (-) refer to ‘not found’.

## Supplementary Materials

The following are available online at https://www.mdpi.com/2223-7747/9/12/1773/s1, Figure S1: The capability of physic nut and sacha inchi rhizobacteria to fix nitrogen, solubilize phosphate and produce siderophore; Figure S2: Morphological characteristics of arbuscular mycorrhizal (AM) spores found in physic nut and sacha inchi rhizosphere soils; Figure S3: A comparison of physic nut plants grown in soils uninoculated and inoculated with their rhizosphere microbes in the pot experiments; Figure S4: A comparison of sacha inchi plants grown in soils uninoculated and inoculated with their rhizosphere microbes in the pot experiments.

## References

[1] Janick J., Paull R.E. (2008). The Encyclopedia of Fruit & Nuts.

[2] Divakara B.N., Upadhyaya H.D., Wani S.P., Gowda C.L. (2010). Biology and genetic improvement of *Jatropha curcas* L.: A review. Appl. Energy.

[3] Koh M.Y., Ghazi T.I.M. (2011). A review of biodiesel production from *Jatropha curcas* L. oil. Renew. Sust. Energy Rev..

[4] Edrisi S.A., Dubey R.K., Tripathi V., Bakshi M., Srivastava P., Jamil S., Singh H.B., Singh N., Abhilash P.C. (2015). *Jatropha curcas* L.: A crucified plant waiting for resurgence. Renew. Sust. Energy Rev..

[5] Lim B.Y., Shamsudin R., Baharudin B.T.H.T., Yunus R. (2015). A review of processing and machinery for *Jatropha curcas* L. fruits and seeds in biodiesel production: Harvesting, shelling, pretreatment and storage. Renew. Sust. Energy Rev..

[6] Kamel D.A., Farag H.A., Amin N.K., Zatout A.A., Ali R.M. (2018). Smart utilization of jatropha (*Jatropha curcas* Linnaeus) seeds for biodiesel production: Optimization and mechanism. Ind. Crops. Prod..

[7] Maes W.H., Trabucco A., Achten W.M.J., Muys B. (2009). Climatic growing conditions of *Jatropha curcas* L.. Biomass Bioenergy.

[8] Achten W.M.J., Maes W.H., Reubens B., Mathijs E., Singh V.P., Verchot L., Muys B. (2010). Biomass production and allocation in *Jatropha curcas* L. seedlings under different levels of drought stress. Biomass Bioenergy.

[9] Silva E.N., Ferreira-Silva S.L., Viégas R.A., Silveira J.A.G. (2010). The role of organic and inorganic solutes in the osmotic adjustment of drought-stressed *Jatropha curcas* plants. Environ. Exp. Bot..

[10] Balota E.L., Machineski O., Viviane Truber P., Scherer A., Souza F.S.D. (2011). Physic nut plants present high mycorrhizal dependency under conditions of low phosphate availability. Braz. J. Plant Physiol..

[11] Zhang C., Zhang L., Zhang S., Zhu S., Wu P., Chen Y., Li M., Jiang H., Wu G. (2015). Global analysis of gene expression profiles in physic nut (*Jatropha curcas* L.) seedlings exposed to drought stress. BMC Plant Biol..

[12] Rawdkuen S., Murdayanti D., Ketnawa S., Phongthai S. (2016). Chemical properties and nutritional factors of pressed-cake from tea and sacha inchi seeds. Food Biosci..

[13] Hanssen H.P., Schmitz-Hübsch M., Preedy V.R., Watson R.R., Patel V.B. (2011). Sacha inchi (*Plukenetia volubilis* L.) nut oil and its therapeutic and nutritional uses. Nuts and Seeds in Health and Disease Prevention.

[14] Kodahl N. (2020). Sacha inchi (*Plukenetia volubilis* L.)—From lost crop of the Incas to part of the solution to global challenges?. Planta.

[15] Zuleta E.C., Rios L.A., Benjumea P.N. (2012). Oxidative stability and cold flow behavior of palm, sacha-inchi, jatropha and castor oil biodiesel blends. Fuel Process. Technol..

[16] Niu L., Tao Y.B., Chen M.S., Fu Q., Li C., Dong Y., Wang X., He H., Xu Z.F. (2016). Selection of reliable reference genes for gene expression studies of a promising oilseed crop, *Plukenetia volubilis*, by real-time quantitative PCR. Int. J. Mol. Sci..

[17] Dutta S., Podile A.R. (2010). Plant growth promoting rhizobacteria (PGPR): The bugs to debug the root zone. Crit. Rev. Microbiol..

[18] Hassan M.K., McInroy J.A., Kloepper J.W. (2019). The interactions of rhizodeposits with plant growth-promoting rhizobacteria in the rhizosphere: A review. Agriculture.

[19] Vacheron J., Desbrosses G., Bouffaud M.L., Touraine B., Moënne-Loccoz Y., Muller D., Legendre L., Wisniewski-Dyé F., Prigent-Combaret C. (2013). Plant growth-promoting rhizobacteria and root system functioning. Front. Plant Sci..

[20] Abdel-Lateif K., Bogusz D., Hocher V. (2012). The role of flavonoids in the establishment of plant roots endosymbioses with arbuscular mycorrhiza fungi, rhizobia and *Frankia* bacteria. Plant Signal. Behav..

[21] Huang X.F., Chaparro J.M., Reardon K.F., Zhang R., Shen Q., Vivanco J.M. (2014). Rhizosphere interactions: Root exudates, microbes, and microbial communities. Botany.

[22] Hugoni M., Luis P., Guyonnet J., el Zahar Haichar F. (2018). Plant host habitat and root exudates shape fungal diversity. Mycorrhiza.

[23] Swamy M.K., Akhtar M.S., Sinniah U.R., Hakeem K.R., Akhtar M.S. (2016). Root exudates and their molecular interactions with rhizospheric microbes. Plant, Soil and Microbes.

[24] Vives-Peris V., de Ollas C., Gómez-Cadenas A., Pérez-Clemente R.M. (2020). Root exudates: From plant to rhizosphere and beyond. Plant Cell Rep..

[25] Sokolova M.G., Akimova G.P., Vaishlya O.B. (2011). Effect of phytohormones synthesized by rhizosphere bacteria on plants. Appl. Biochem. Microbiol..

[26] Kang S.M., Khan A.L., Hamayun M., Hussain J., Joo G.J., You Y.H., Kim J.G., Lee I.J. (2012). Gibberellin-producing *Promicromonospora* sp. SE188 improves *Solanum lycopersicum* plant growth and influences endogenous plant hormones. J. Microbiol..

[27] Shi T.Q., Peng H., Zeng S.Y., Ji R.Y., Shi K., Huang H., Ji X.J. (2017). Microbial production of plant hormones: Opportunities and challenges. Bioengineered.

[28] Kudoyarova G.R., Arkhipova T.N., Melent’ev A.I., Maheshwari D.K. (2015). Role of bacterial phytohormones in plant growth regulation and their development. Bacterial Metabolites in Sustainable Agroecosystem.

[29] Chaiharn M., Lumyong S. (2009). Phosphate solubilization potential and stress tolerance of rhizobacteria from rice soil in Northern Thailand. World J. Microbiol. Biotechnol..

[30] Kuan K.B., Othman R., Abdul Rahim K., Shamsuddin Z.H. (2016). Plant growth-promoting rhizobacteria inoculation to enhance vegetative growth, nitrogen fixation and nitrogen remobilisation of maize under greenhouse conditions. PLoS ONE.

[31] Fan X., Zhang S., Mo X., Li Y., Fu Y., Liu Z. (2017). Effects of plant growth-promoting rhizobacteria and N source on plant growth and N and P uptake by tomato grown on calcareous soils. Pedosphere.

[32] Ghavami N., Alikhani H.A., Pourbabaei A.A., Besharati H. (2017). Effects of two new siderophore-producing rhizobacteria on growth and iron content of maize and canola plants. J. Plant Nutr..

[33] Manzoor M., Abbasi M.K., Sultan T. (2017). Isolation of phosphate solubilizing bacteria from maize rhizosphere and their potential for rock phosphate solubilization–mineralization and plant growth promotion. Geomicrobiol. J..

[34] Xie X., Weng B., Cai B., Dong Y., Yan C. (2014). Effects of arbuscular mycorrhizal inoculation and phosphorus supply on the growth and nutrient uptake of *Kandelia obovata* (Sheue, Liu & Yong) seedlings in autoclaved soil. Appl. Soil Ecol..

[35] Battini F., Grønlund M., Agnolucci M., Giovannetti M., Jakobsen I. (2017). Facilitation of phosphorus uptake in maize plants by mycorrhizosphere bacteria. Sci. Rep..

[36] Chaiyasen A., Douds D.D., Gavinlertvatana P., Lumyong S. (2017). Diversity of arbuscular mycorrhizal fungi in *Tectona grandis* Linn.f. plantations and their effects on growth of micropropagated plantlets. New For..

[37] Chen S., Zhao H., Zou C., Li Y., Chen Y., Wang Z., Jiang Y., Liu A., Zhao P., Wang M. (2017). Combined inoculation with multiple arbuscular mycorrhizal fungi improves growth, nutrient uptake and photosynthesis in cucumber seedlings. Front. Microbiol..

[38] Wang Y., Wang M., Li Y., Wu A., Huang J. (2018). Effects of arbuscular mycorrhizal fungi on growth and nitrogen uptake of *Chrysanthemum morifolium* under salt stress. PLoS ONE.

[39] Ingraffia R., Amato G., Frenda A.S., Giambalvo D. (2019). Impacts of arbuscular mycorrhizal fungi on nutrient uptake, N_2_ fixation, N transfer, and growth in a wheat/faba bean intercropping system. PLoS ONE.

[40] Porcel R., Aroca R., Ruiz-Lozano J.M. (2012). Salinity stress alleviation using arbuscular mycorrhizal fungi: A review. Agron. Sustain. Dev..

[41] Wu Q.S., Srivastava A.K., Zou Y.N. (2013). AMF-induced tolerance to drought stress in citrus: A review. Sci. Hortic..

[42] Balliu A., Sallaku G., Rewald B. (2015). AMF inoculation enhances growth and improves the nutrient uptake rates of transplanted, salt-stressed tomato seedlings. Sustainability.

[43] Spagnoletti F.N., Balestrasse K., Lavado R.S., Giacometti R. (2016). Arbuscular mycorrhiza detoxifying response against arsenic and pathogenic fungus in soybean. Ecotox. Environ. Saf..

[44] Begum N., Qin C., Ahanger M.A., Raza S., Khan M.I., Ashraf M., Ahmed N., Zhang L. (2019). Role of arbuscular mycorrhizal fungi in plant growth regulation: Implications in abiotic stress tolerance. Front. Plant Sci..

[45] Gao X., Lu X., Wu M., Zhang H., Pan R., Tian J., Li S., Liao H. (2012). Co-inoculation with rhizobia and AMF inhibited soybean red crown rot: From field study to plant defense-related gene expression analysis. PLoS ONE.

[46] Hernández-Montiel L.G., Rueda-Puente E.O., Cordoba-Matson M.V., Holguín-Peña J.R., Zulueta-Rodríguez R. (2013). Mutualistic interaction of rhizobacteria with arbuscular mycorrhizal fungi and its antagonistic effect on *Fusarium oxysporum* in *Carica papaya* seedlings. Crop Prot..

[47] Li H., Wang C., Li X., Xiang D. (2013). Inoculating maize fields with earthworms (*Aporrectodea trapezoides*) and an arbuscular mycorrhizal fungus (*Rhizophagus intraradices*) improves mycorrhizal community structure and increases plant nutrient uptake. Biol. Fert. Soils.

[48] Svenningsen N.B., Watts-Williams S.J., Joner E.J., Battini F., Efthymiou A., Cruz-Paredes C., Nybroe O., Jakobsen I. (2018). Suppression of the activity of arbuscular mycorrhizal fungi by the soil microbiota. ISME J..

[49] Jha C.K., Patel D., Rajendran N., Saraf M. (2010). Combinatorial assessment on dominance and informative diversity of PGPR from rhizosphere of *Jatropha curcas* L.. J. Basic Microbiol..

[50] Jha C.K., Saraf M. (2012). Evaluation of multispecies plant-growth-promoting consortia for the growth promotion of *Jatropha curcas* L.. J. Plant Growth Regul..

[51] Jha C.K., Annapurna K., Saraf M. (2011). Isolation of rhizobacteria from *Jatropha curcas* and characterization of produced ACC deaminase. J. Basic Microbiol..

[52] Jha C.K., Patel B., Saraf M. (2012). Stimulation of the growth of *Jatropha curcas* by the plant growth promoting bacterium *Enterobacter cancerogenus* MSA2. World J. Microbiol. Biotechnol..

[53] Madhaiyan M., Peng N., Te N.S., Hsin I.C., Lin C., Lin F., Reddy C., Yan H., Ji L. (2013). Improvement of plant growth and seed yield in *Jatropha curcas* by a novel nitrogen-fixing root associated *Enterobacter* species. Biotechnol. Biofuels.

[54] Natarajan A., Kumar K., Madhuri K., Usharani G.K. (2016). Isolation and characterization of salt tolerant plant growth promoting rhizobacteria from plants grown in Tsunami affected regions of Andaman and Nicobar Islands. Geomicrobiol. J..

[55] Charoenpakdee S., Cherdchai P., Dell B., Lumyong S. (2010). The mycorrhizal status of indigenous arbuscular mycorrhizal fungi of physic nut *Jatropha curcas* in Thailand. Mycosphere.

[56] Charoenpakdee S., Phosri C., Dell B., Choonluechanon S. (2010). Compatible arbuscular mycorrhizal fungi of *Jatropha curcas* and spore multiplication using cereal crops. Mycosphere.

[57] Kumar A., Sharma S., Mishra S., Dames J.F. (2013). Arbuscular mycorrhizal inoculation improves growth and antioxidative response of *Jatropha curcas* (L.) under Na_2_SO_4_ salt stress. Plant Biosyst..

[58] Patel D., Saraf M. (2013). Influence of soil ameliorants and microflora on induction of antioxidant enzymes and growth promotion of *Jatropha curcas* L. under saline condition. Eur. J. Soil Biol..

[59] Kumar A., Sharma S., Mishra S. (2015). Evaluating effect of arbuscular mycorrhizal fungal consortia and *Azotobacter chroococcum* in improving biomass yield of *Jatropha curcas*. Plant Biosyst..

[60] Corazon-Guivin M.A., Cerna-Mendoza A., Guerrero-Abad J.C., Vallejos-Tapullima A., da Silva G.A., Oehl F. (2019). *Acaulospora aspera*, a new fungal species in the *Glomeromycetes* from rhizosphere soils of the Inka nut (*Plukenetia volubilis* L.) in Peru. J. Appl. Bot. Food Qual..

[61] Corazon-Guivin M.A., Mendoza A.C., Guerrero-Abad J.C., Vallejos-Tapullima A., Carballar-Hernández S., da Silva G.A., Oehl F. (2019). *Funneliglomus*, gen. nov., and *Funneliglomus sanmartinensis*, a new arbuscular mycorrhizal fungus from the Amazonia region in Peru. Sydowia.

[62] Corazon-Guivin M.A., Cerna-Mendoza A., Guerrero-Abad J.C., Vallejos-Tapullima A., Carballar-Hernández S., da Silva G.A., Oehl F. (2019). *Microkamienskia* gen. nov. and *Microkamienskia peruviana*, a new arbuscular mycorrhizal fungus from Western Amazonia. Nova Hedwig.

[63] Wang S., Chen X., Gong H., Cai Z. (2018). Response of soil microbial abundance and diversity in Sacha Inchi (*Plukenetia volubilis* L.) farms with different land-use histories in a tropical area of Southwestern China. Arch. Agrono Soil Sci..

[64] Tian Y.H., Lei Y.B., Zheng Y.L., Cai Z.Q. (2013). Synergistic effect of colonization with arbuscular mycorrhizal fungi improves growth and drought tolerance of *Plukenetia volubilis* seedlings. Acta Physiol. Plant.

[65] Kennedy A.C., de Luna L.Z., Hillel D., Hatfield J.L. (2005). Rhizosphere. Encyclopedia of Soils in the Environment.

[66] Park M., Kim C., Yang J., Lee H., Shin W., Kim S., Sa T. (2005). Isolation and characterization of diazotrophic growth promoting bacteria from rhizosphere of agricultural crops of Korea. Microbiol. Res..

[67] Nakaew N., Rangjaroen C., Sungthong R. (2015). Utilization of rhizospheric *Streptomyces* for biological control of *Rigidoporus* sp. causing white root disease in rubber tree. Eur. J. Plant Pathol..

[68] Kumla J., Suwannarach N., Bussaban B., Matsui K., Lumyong S. (2014). Indole-3-acetic acid production, solubilization of insoluble metal minerals and metal tolerance of some sclerodermatoid fungi collected from northern Thailand. Ann. Microbiol..

[69] Rangjaroen C., Rerkasem B., Teaumroong N., Noisangiam R., Lumyong S. (2014). Promoting plant growth in a commercial rice cultivar by endophytic diazotrophic bacteria isolated from rice landraces. Ann. Microbiol..

[70] Prakamhang J., Minamisawa K., Teamtaisong K., Boonkerd N., Teaumroong N. (2009). The communities of endophytic diazotrophic bacteria in cultivated rice (*Oryza sativa* L.). Appl. Soil Ecol..

[71] Desvaux M., Guedon E., Petitdemange H. (2000). Cellulose catabolism by *Clostridium cellulolyticum* growing in batch culture on defined medium. Appl. Environ. Microbiol..

[72] Wu Q.S., Xia R.X. (2006). Arbuscular mycorrhizal fungi influence growth, osmotic adjustment and photosynthesis of citrus under well-watered and water stress conditions. J. Plant Physiol..

[73] Lane D.J., Stackebrandt E., Goodfellow M. (1991). 16S/23S rRNA sequencing. Nucleic Acid Techniques in Bacterial Systematics.

[74] Xiang D., Chen B., Li H. (2016). Specificity and selectivity of arbuscular mycorrhizal fungal polymerase chain reaction primers in soil samples by clone library analyses. Acta Agric. Scand..

[75] White T.J., Bruns T., Lee S., Taylor J.W., Innis M.A., Gelfand D.H., Sninsky J.J., White T.J. (1990). Amplification and direct sequencing of fungal ribosomal RNA genes for phylogenetics. PCR Protocols A Guide to Methods and Applications.

[76] Lee J., Lee S., Young J.P.W. (2008). Improved PCR primers for the detection and identification of arbuscular mycorrhizal fungi. FEMS Microbiol. Ecol..

