# Supplementary material for: Rhizobacteria and Arbuscular Mycorrhizal Fungi of Oil Crops (Physic Nut and Sacha Inchi): A Cultivable-Based Assessment for Abundance, Diversity, and Plant Growth-Promoting Potentials
Source: Plants (Basel). 2020 Dec 14;9(12):1773. doi: 10.3390/plants9121773 (PMC7765041; doi:10.3390/plants9121773)
Supplement: Supplementary file 1 [file plants-09-01773-s001.pdf]

plants

MDPI

Supplementary Materials

Rhizobacteria and Arbuscular Mycorrhizal Fungi of Oil Crops (Physic Nut and Sacha Inchi): Abundance, Diversity and Plant Growth-Promoting Potentials

Janjira Wiriya [1,2], Chakrapong Rangjaroen [3], Neung Teaumroong [4], Rungroch Sungthong [5,*] and Saisamorn Lumyong [1,6,7,*]

[1] Department of Biology, Faculty of Science, Chiang Mai University, Chiang Mai 50200, Thailand; janjira.wiriya209@gmail.com (J.W.); scboi009@gmail.com (S.L.)
[2] Graduate School, Chiang Mai University, Chiang Mai 50200, Thailand; janjira.wiriya209@gmail.com
[3] Department of Agricultural Technology, Faculty of Science and Technology, Phranakhon Rajabhat University, Bangkok 10220, Thailand; rangjaroen@gmail.com
[4] School of Biotechnology, Institute of Agricultural Technology, Suranaree University of Technology, Nakhon Ratchasima 30000, Thailand; neung@sut.ac.th
[5] Laboratory of Hydrology and Geochemistry of Strasbourg, University of Strasbourg, UMR 7517 CNRS/EOST, Strasbourg Cedex 67084, France; sungthong@unistra.fr
[6] Center of Excellence in Microbial Diversity and Sustainable Utilization, Faculty of Science, Chiang Mai University, Chiang Mai 50200, Thailand; scboi009@gmail.com
[7] Academy of Science, The Royal Society of Thailand, Bangkok 10300, Thailand; scboi009@gmail.com
* Correspondence: sungthong@unistra.fr (R.S.), scboi009@gmail.com (S.L.)

Figure S1. The capability of physic nut and sacha inchi rhizobacteria to fix nitrogen, solubilize phosphate and produce siderophore. Only the top three excellent rhizobacterial isolates per cultivation site are presented. The origins of these bacteria are available in Table 2.

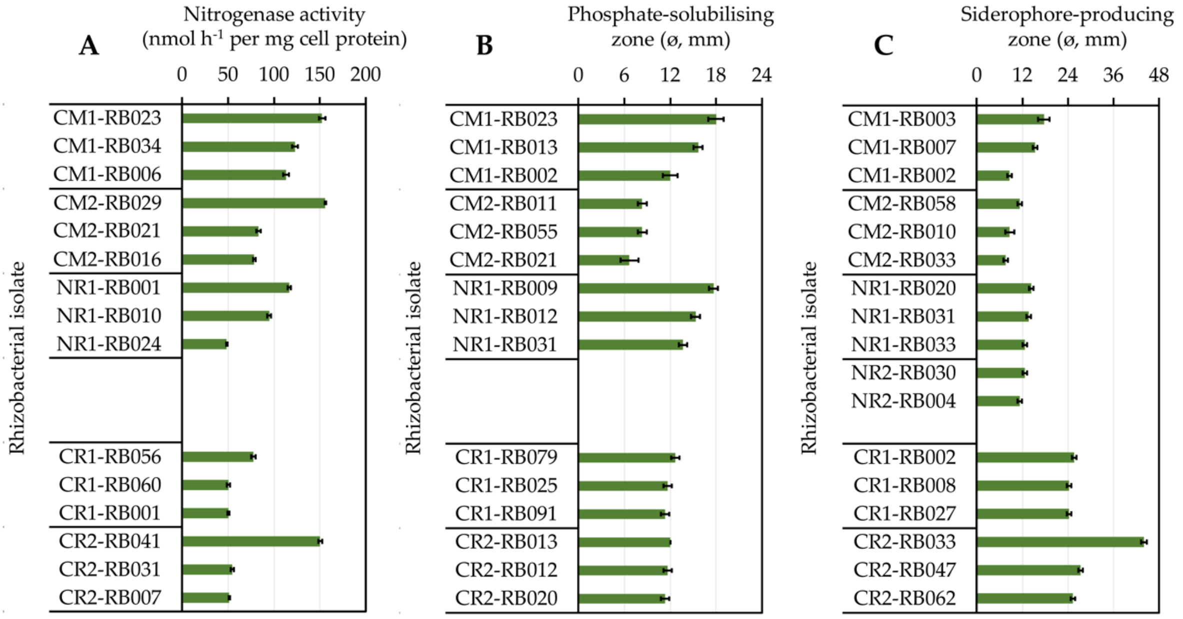

Figure S2. Morphological characteristics of arbuscular mycorrhizal (AM) spores found in physic nut and sacha inchi rhizosphere soils. Groups A1 – A9 represent the different spore features of the genus *Acaulospora*, while groups C1, G1 and G2, and F1 exhibit the unique spore characteristics of the genera *Claroideoglomus*, *Glomus*, and *Funneliformis*, respectively. Scale bars = 100 µm.

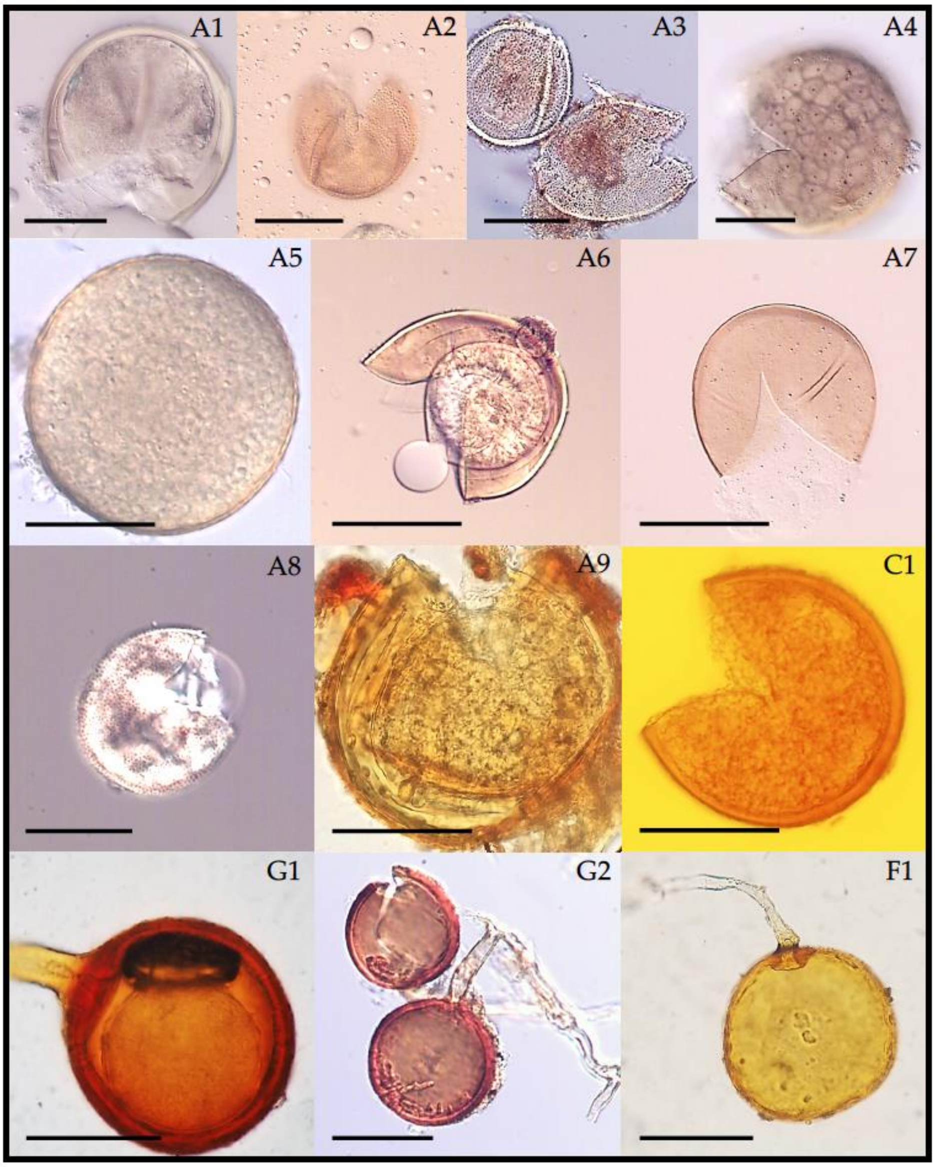

Figure S3. A comparison of physic nut plants grown in soils uninoculated and inoculated with their rhizosphere microbes in the pot experiments. The treatments include T1: untreated control, T2: inoculated with a rhizobacterium *Ensifer* sp. CM1-RB003, T3: inoculated with an AM fungus *Acaulospora* sp. CM2-AMA3, and T4: inoculated with a mixture of *Ensifer* sp. CM1-RB003 and *Acaulospora* sp. CM2-AMA3. Scale bars = 10 cm. The origins of these rhizosphere microbes are available in Tables 2, 3 and 4, and the experimental description can be found in the text.

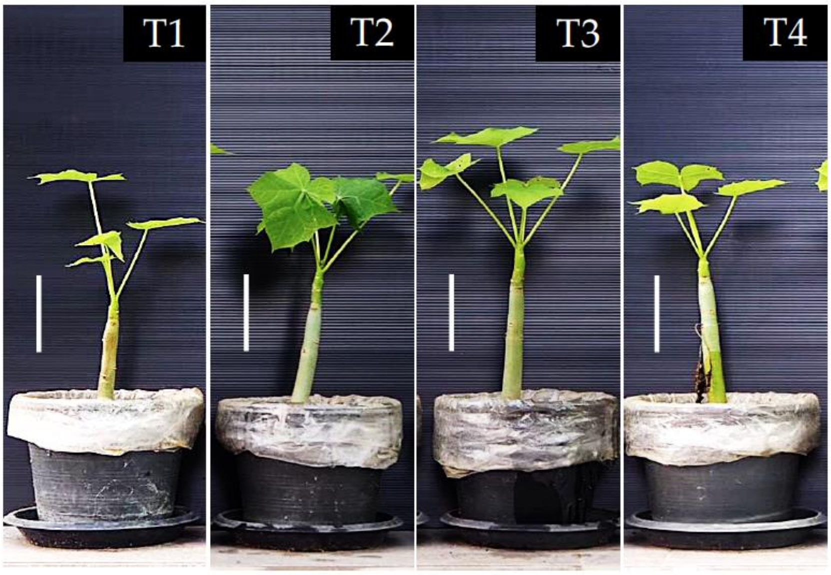

Figure S4. A comparison of sacha inchi plants grown in soils uninoculated and inoculated with their rhizosphere microbes in the pot experiments. The treatments include T1: untreated control, T2: inoculated with a rhizobacterium *Pantoea* sp. CR1-RB056, T3: inoculated with an AM fungus *Funneliformis* sp. CR2-AMF1, and T4: inoculated with a mixture of *Pantoea* sp. CR1-RB056 and *Funneliformis* sp. CR2-AMF1. Scale bars = 25 cm. The origins of these rhizosphere microbes are available in Tables 2, 3 and 4, and the experimental description can be found in the text.

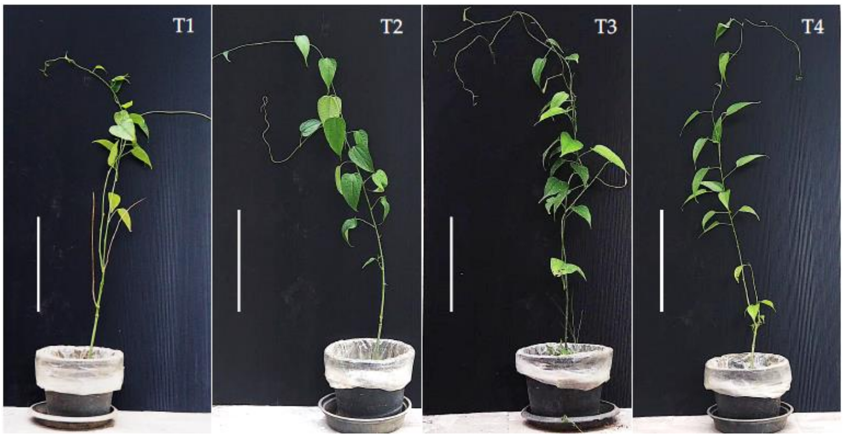

© 2020 by the authors. Submitted for possible open access publication under the terms and conditions of the Creative Commons Attribution (CC BY) license (http://creativecommons.org/licenses/by/4.0/).
